# Dynamic Behaviors of Solvent Molecules Restricted in Poly (Acryl Amide) Gels Analyzed by Dielectric and Diffusion NMR Spectroscopy

**DOI:** 10.3390/gels4030056

**Published:** 2018-06-22

**Authors:** Hironobu Saito, Shunpei Kato, Keisuke Matsumoto, Yuya Umino, Rio Kita, Naoki Shinyashiki, Shin Yagihara, Minoru Fukuzaki, Masayuki Tokita

**Affiliations:** 1 Graduate School of Science and Technology, Tokai University, Kanagawa 259-1292, Japan; hilonobu.saito@gmail.com; 2 Graduate School of Science, Tokai University, 4-1-1 Kitakaname, Hiratsuka-shi, Kanagawa 259-1292, Japan; chikyuunimoyasasiku@gmail.com (S.K.); se2gekka@gmail.com (K.M.); yuno_estrade@ezweb.ne.jp (Y.U.); rkita@keyaki.cc.u-tokai.ac.jp (R.K.); naoki-ko@keyaki.cc.u-tokai.ac.jp (N.S.); 3 Liberal Arts Education Center in Kumamoto, Tokai University, Toroku 9-1-1, Higashi-ku, Kumamoto-shi, Kumamoto 862-0970, Japan; mfukuzaki@tokai-u.jp; 4 Graduate School of Science, Kyushu University, 744, Motooka, Nishi-ku, Fukuoka 819-0395, Japan; tokita@phys.kyushu-u.ac.jp

**Keywords:** poly (acryl amide) gel, time domain reflectometry (TDR) of dielectric spectroscopy, pulse field gradient spin echo method of nuclear magnetic resonance (PFG-NMR), scaling analysis, fractal analysis

## Abstract

Dynamics of solvent molecules restricted in poly (acryl amide) gels immersed in solvent mixtures of acetone–, 1,4-dioxane–, and dimethyl sulfoxide–water were analyzed by the time domain reflectometry method of dielectric spectroscopy and the pulse field gradient method of nuclear magnetic resonance. Restrictions of dynamic behaviors of solvent molecules were evaluated from relaxation parameters such as the relaxation time, its distribution parameter, and the relaxation strength obtained by dielectric measurements, and similar behaviors with polymer concentration dependences for the solutions were obtained except for the high polymer concentration in collapsed gels. Scaling analyses for the relaxation time and diffusion coefficient respectively normalized by those for bulk solvent suggested that the scaling exponent determined from the scaling variable defined as a ratio of the size of solvent molecule to mesh size of polymer networks were three and unity, respectively, except for collapsed gels. The difference in these components reflects characteristic molecular interactions in the rotational and translational diffusions, and offered a physical picture of the restriction of solvent dynamics. A universal treatment of slow dynamics due to the restriction from polymer chains suggests a new methodology of characterization of water structures.

## 1. Introduction

Polymer gels, i.e., crosslinked polymer networks with solvent have been extensively applied in diverse fields such as medical, environment, and food science and industries because of the characteristic behaviors. One of the most typical and characteristic behaviors of polymer gels is a volume phase transition with which the late Professor Tanaka has first indicated that fundamental physics of polymer gels, leading to expanded usage in applied science. Poly (acryl amide) (PAAm) gels swell and shrink sensitively with a change in environment, such as temperature, electric field, composition of solvent, ion concentration, pH, etc. [1,2,3,4,5,6]. However, there still exist some ambiguities in physical properties of the polymer gel related to the volume phase transition, especially from a view point of molecular interactions among the solvent molecules and polymer chains.

One of the authors (M.T.) has reported that diffusion coefficients of probe molecules obtained by the pulsed field gradient spin echo method of nuclear magnetic resonance (PFG-NMR) [7,8] showed dynamic behaviors of probe molecules restricted in polymer gels [9,10,11]. The mesh size of the network could be evaluated from the restriction of various probe molecules with different sizes. This methodology can be an effective tool for the evaluation of gels of not only synthetic but also natural polymers [12,13]. However, this method generally requires some probe molecules, since water protons cannot be used with the recent high-resolution equipment because of oscillation dumping. There has not been a lot of techniques to evaluate the volume phase transition without addition of probe molecules.

We have investigated the dynamic behavior of solvent molecules restricted in polymer networks of gels [14,15] by using the time domain reflectometry (TDR) method [16,17,18] of microwave dielectric spectroscopy. The TDR method is capable of observing relaxation processes in the GHz frequency region including the most important frequencies for liquid molecules at ordinary temperatures and those slow dynamics with restrictions in polymer gels. Furthermore, open-end coaxial electrodes, which can be attached to the surface of gel and solid materials for dielectric measurements with the fringing field, are easily used in TDR measurements [14]. Therefore, in the case of dielectric studies on the volume phase transition, the TDR measuring system is not simply a conventional method, and various refinements makes the TDR method the most effective tool for detailed analysis and discussion, especially for cooperative dynamics of mixed solvent.

Recently, Yang and Zhao, et al. [19,20,21], reported dielectric properties of the temperature-sensitive phase transition of poly (*N*-isopropylacrylamide) (PNiPAM)-based microgels, for which conventional electrodes are available. The PNiPAM gel is also typical polymer gels expected to be applied for medial usages like microcapsules and drug delivery systems. Relaxation processes observed at frequencies lower than 110 MHz are of interest in chain dynamics, however, solvent dynamics and behaviors are not directly reflected in the frequency region.

In the present work, we examined acetone, 1,4-dioxane, and dimethyl sulfoxide (DMSO) as organic solvents of aqueous mixtures with various compositions, in order to investigate the molecular mechanism bringing abrupt volume changes of PAAm gels with the composition of the organic solvent–water mixtures. PFG-NMR and TDR measurements were performed to compare dynamic behaviors of solvent molecules inside gels with those for bulk liquid mixtures. Various difficulties, such as the contact of TDR electrodes with samples, high frequency measurements of water, and oscillation dumping for water protons in TDR and NMR measuring techniques for the gel study require more creative treatments of the equipment. Furthermore, the complementarily analysis with the characteristic time scales of 1 ms for NMR and 10 ps for TDR measurements offers us more detailed analysis of the fluctuation of the order parameters, such as the relaxation time distributions. The three organic solvents were chosen because of their different characteristics: 1,4-dioxane is a non-polar liquid; acetone and DMSO have a similar chemical structure. Each component of the solvent mixtures respectively interacts with polymer chains with different affinity. The differences in their dynamics reflected the restricted behavior of these solvent molecules and led us to more detailed analysis of the intermolecular interactions among solvent molecules and polymer chains.

## 2. Results and Discussion

### 2.1. Swelling Ratio of Gels

**Figure 1:**
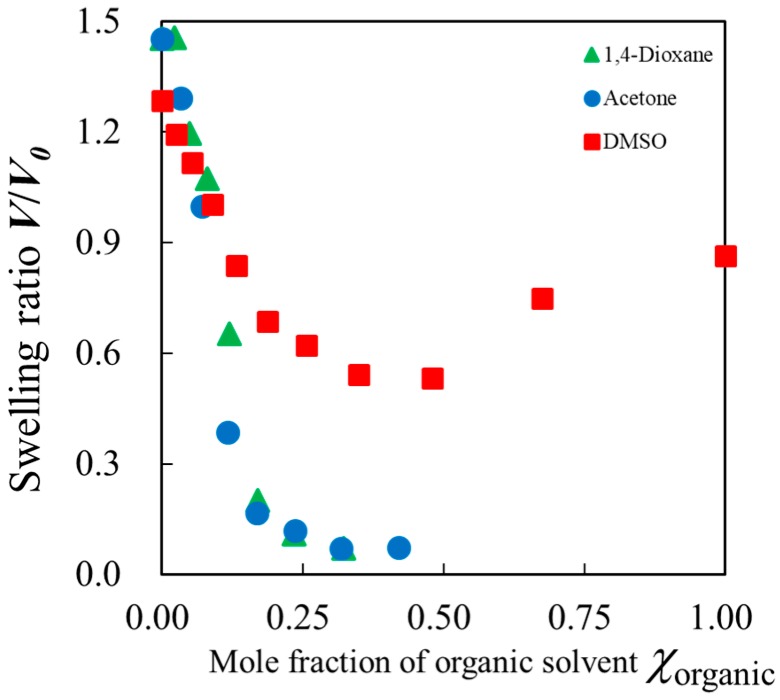
The swelling ratio for poly (acryl amide) (PAAm) gel normalized by the initial value in water, *V*/*V*_0_, dependent on the mole fraction of organic solvent in the aqueous solution for acetone, 1,4-dioxane, and dimethyl sulfoxide (DMSO).

Figure 1 shows mole fraction dependences of the swelling ratio, *V*/*V*_0_, for PAAm gels immersed in aqueous mixtures of acetone, 1,4-dioxiane, and DMSO, respectively, with some concentrations raised step by step to reach an equilibrium volume at the target concentration. Here, *V* was determined from the equilibrium volume of the gel at certain composition of the solvent mixtures and *V*_0_ is the initial volume of the gel prepared in pure water. The initial volume, *V*_0_, was determined when the gels were synthesized at the polymer concentration of about 5.1 wt %. Upon immersing the initial gel in pure water, the gel swelled and reached the equilibrium volume. Then, the value, *V*/*V*_0_, was larger than unity in water. The result for the acetone–water mixtures shows an abrupt change in the mole fraction region of 0.1 < *χ*_organic_ < 0.2 as reported by Tanaka et al. [1]. A similar result was also obtained for the gel immersed in 1,4-dioxane aqueous solution. On the other hand, the PAAm gel with DMSO aqueous solution showed a similar but a smaller volume change and an opposite volume change appeared in the DMSO-rich region.

### 2.2. PFG-NMR Measurements

**Figure 2:**
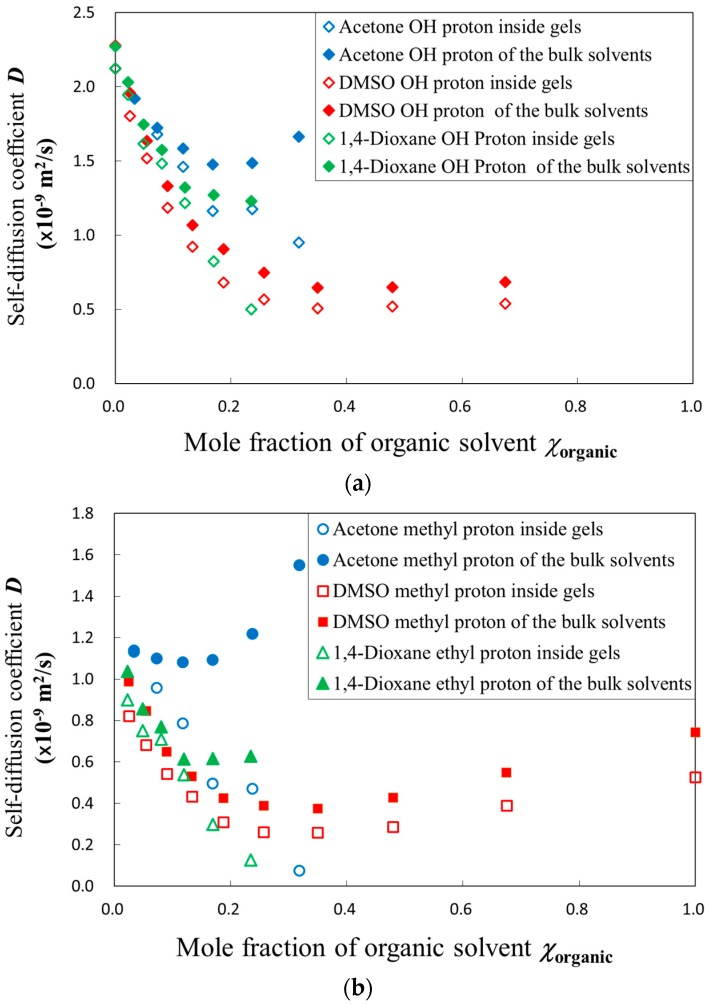
Acetone and DMSO mole fraction dependence of the diffusion coefficient for solvent molecules inside gel or bulk solvent obtained from: (**a**) Proton of water molecules; (**b**) Methyl proton of organic solvent molecules.

Usual application of the PFG-NMR method to gel materials requires probe molecules [9,11,13]. We have reported that liquid structures, including hydrogen bonding networks, reflect larger scales of interactions [22,23]. Thus, protons of water and organic solvent molecules were used to examine the molecular diffusion processes in the present work. Figure 2a shows acetone and DMSO mole fraction, *χ*_organic_, dependences of the diffusion coefficients for water molecules of the solvent mixtures inside gels and the bulk solvent obtained from PFG-NMR method.

All plots decreased with increasing mole fraction of organic solvents in the region, *χ*_organic_ < 0.2. The diffusion coefficient of solvent molecules restricted in the gels were smaller than those in the bulk solvents. However, composition dependences of the diffusion coefficient for DMSO were different from those for acetone. Former dielectric studies on aqueous solutions of organic solvent usually showed changes in composition dependences of relaxation parameters at around the mole fraction of water, 0.83, even if those changes are only apparent [24,25]. Actually, our results on the diffusion coefficient for the present solvent mixtures also apparently indicated changes in the composition dependences as shown at around the mole fraction of the organic solvent, 0.17. The solvent mixtures take characteristic values of the diffusion coefficient in the region, χ_organic_ > 0.2, as shown in Figure 2a.

Figure 2b shows similar results of the restrictions of dynamic behaviors also for the organic solvent molecules. The smaller values of the diffusion coefficient for the organic solvents than those for water molecules mean more restrictions because of the larger size of molecules. It is available even in the case of solvent mixtures without gels. Furthermore, comparing with the size of solvent molecules, the mesh size is generally large enough, since the ratio of the tetrafunctional monomer used for crosslinking was just 1/20 against the bifunctional monomers for chains. Therefore, the restrictions were almost entirely from polymer chains and the effect of shrinking is accompanied with increasing polymer concentrations. The restriction we compared among the organic solvents indicated the solvent size dependency in the diffusion coefficient.

**Figure 3:**
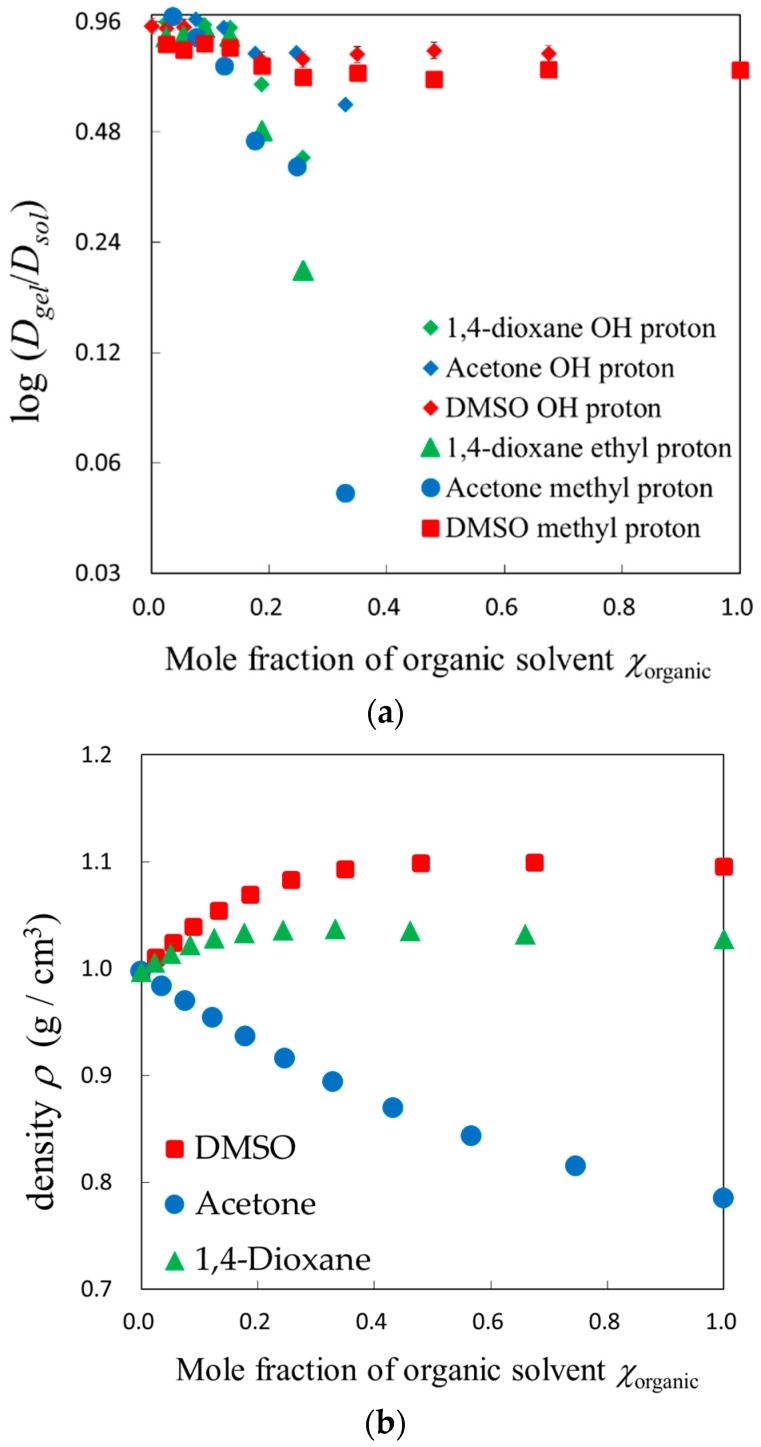
Mole fraction of organic solvent dependences of the diffusion coefficient and density for each solvent mixture: (**a**) The diffusion coefficient of solvent in gels normalized by those in bulk solvent; (**b**) The density of bulk solvent mixtures.

In order to focus attention on the restriction from the polymer chain networks in the gels, the diffusion coefficient of solvent molecules in the gels was normalized by those obtained in the bulk solvent mixtures. Figure 3a shows plots of the logarithm of the normalized diffusion coefficient, *D_gel_*/*D_sol_*, against the composition of solvent mixtures for acetone, 1,4-dioxane, and DMSO aqueous solutions. The normalized diffusion coefficient for acetone and 1,4-dioxane became smaller with shrinking gels, but DMSO did not show similar behavior, since the swelling ratio of gels for DMSO aqueous solutions was too large to restrict solvent dynamics. The same explanation is available for larger values of the normalized diffusion coefficient for water molecules. The composition dependence of the normalized diffusion coefficient for 1,4-dioxane aqueous solution was similar to the acetone aqueous solution as their swelling ratio behaviors were also similar. Figure 3b shows composition dependences of density for the organic solvent aqueous solutions. Characteristic properties of density for each organic solvent’s aqueous solution were not directly reflected in the dynamic behaviors, since the dynamics restricted by polymer chain networks were affected more by the swelling ratio.

### 2.3. TDR Meserments

**Figure 4:**
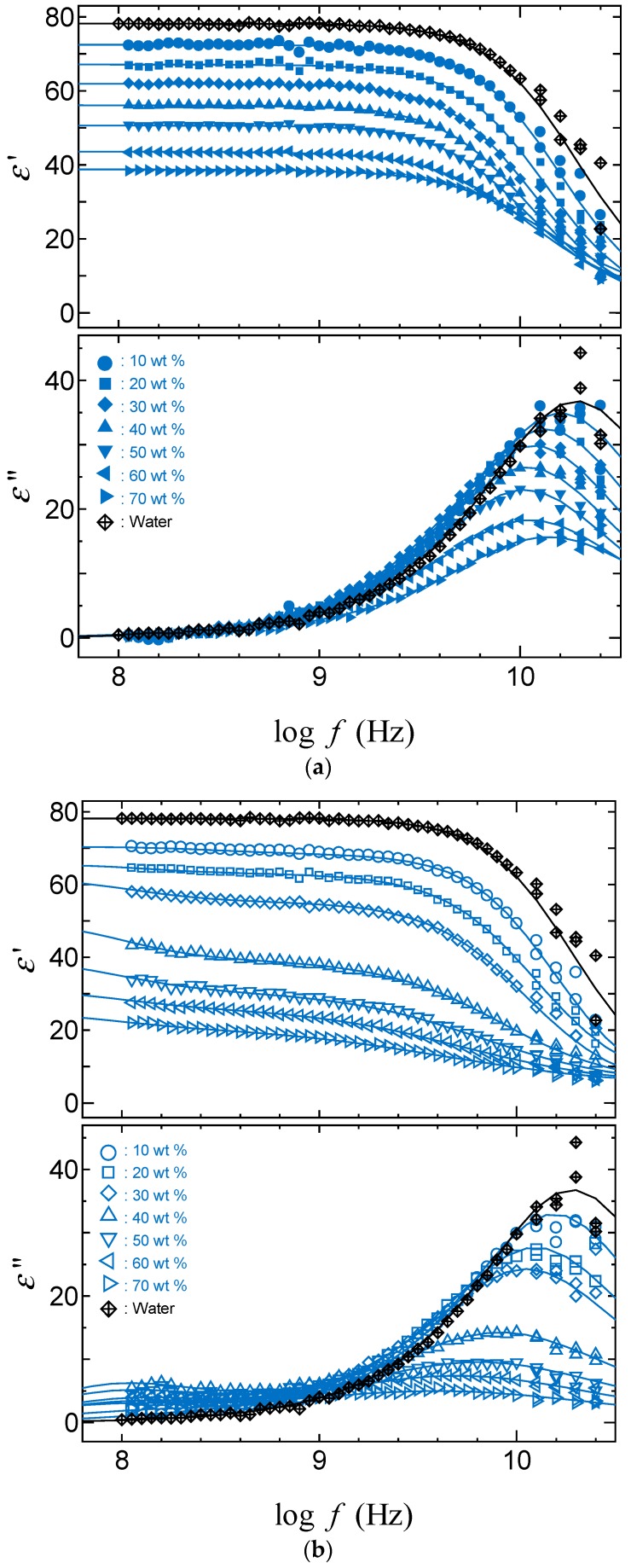
Dielectric dispersion and absorption curves for acetone–water mixtures: (**a**) Outside PAAm gels; (**b**) Inside PAAm gels.

**Figure 5:**
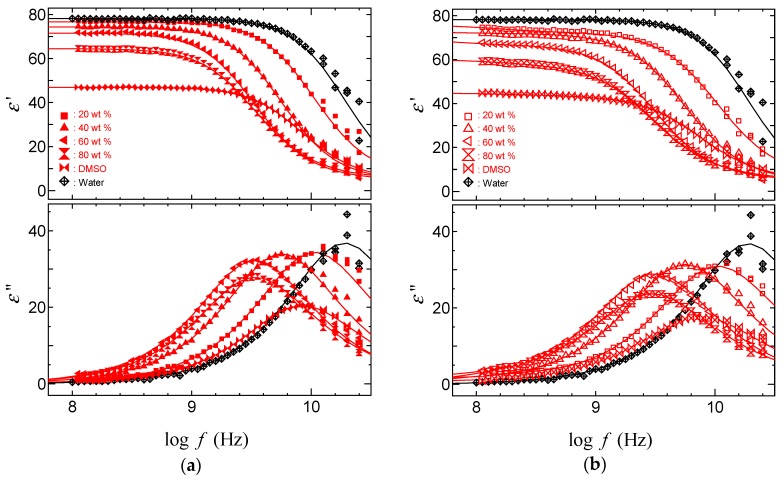
Dielectric dispersion and absorption curves for DMSO–water mixtures: (**a**) Outside PAAm gels; (**b**) Inside PAAm gels.

Figure 4 and Figure 5 show dielectric dispersion and absorption curves obtained by TDR measurements for acetone and DMSO aqueous solutions with PAAm gels. The binary mixtures of polar molecules show only one relaxation process, even if these diffusion coefficients show two values. This is also an indication of an averaging effect of the dielectric properties with the large-scale behaviors of hydrogen bonding liquids. Restrictions of the dynamic behaviors appear as a lower frequency shift of the peak frequency and a decrease in the relaxation strength. Differences between the curves for the solvent inside and outside the gel is larger in the water poor region with smaller swelling ratio. Then, comparing Figure 4 and Figure 5, the larger differences between solvent molecules inside and outside the gels shown for acetone aqueous solutions are simply explained from a larger decrease in the swelling ratio. The difference indicating the restriction of molecular dynamics of solvent in the polymer network is characterized by a lower frequency shift of the peak frequency and a smaller relaxation process with increasing polymer concentration with the shrinkage of gels.

The relaxation parameters were obtained from the fitting procedures to dielectric relaxation data with the following Equation,

(1) $$\varepsilon^{*}=\varepsilon \prime -j\varepsilon \prime \prime =\varepsilon_{\infty }+\frac{\Delta \varepsilon }{{\left[1+{\left(j\omega \tau \right)}^{\beta }\right]}^{\alpha }}-j\frac{\sigma_{\mathrm{DC}}}{\varepsilon_{0}\omega }$$

where *ε*′ and *ε*″ are the real and imaginary parts of the complex dielectric constant, ε*, respectively, *j* is the imaginary unit, *ε*_∞_ is the limiting high-frequency dielectric constant, *∆ε* is the relaxation strength, *ω* is the angular frequency, *τ* is the relaxation time, *α* and *β* (0 < *α*, *β ≤* l) is the relaxation time distribution parameter, and *σ*_DC_ is the dc conductivity, *ε*_0_ is the dielectric constant of vacuum. The relaxation function used for the fitting procedure was Cole–Cole Equation (*α* = l) [26] for 1,4-dioxane and acetone aqueous solutions, Cole-Davidson Equation (*β* = l) [27] for bulk DMSO aqueous solutions [28,29], and Havriliak–Negami Equation (0 < *α*, *β ≤* l) [30] for DMSO aqueous solutions inside gels. 

**Figure 6:**
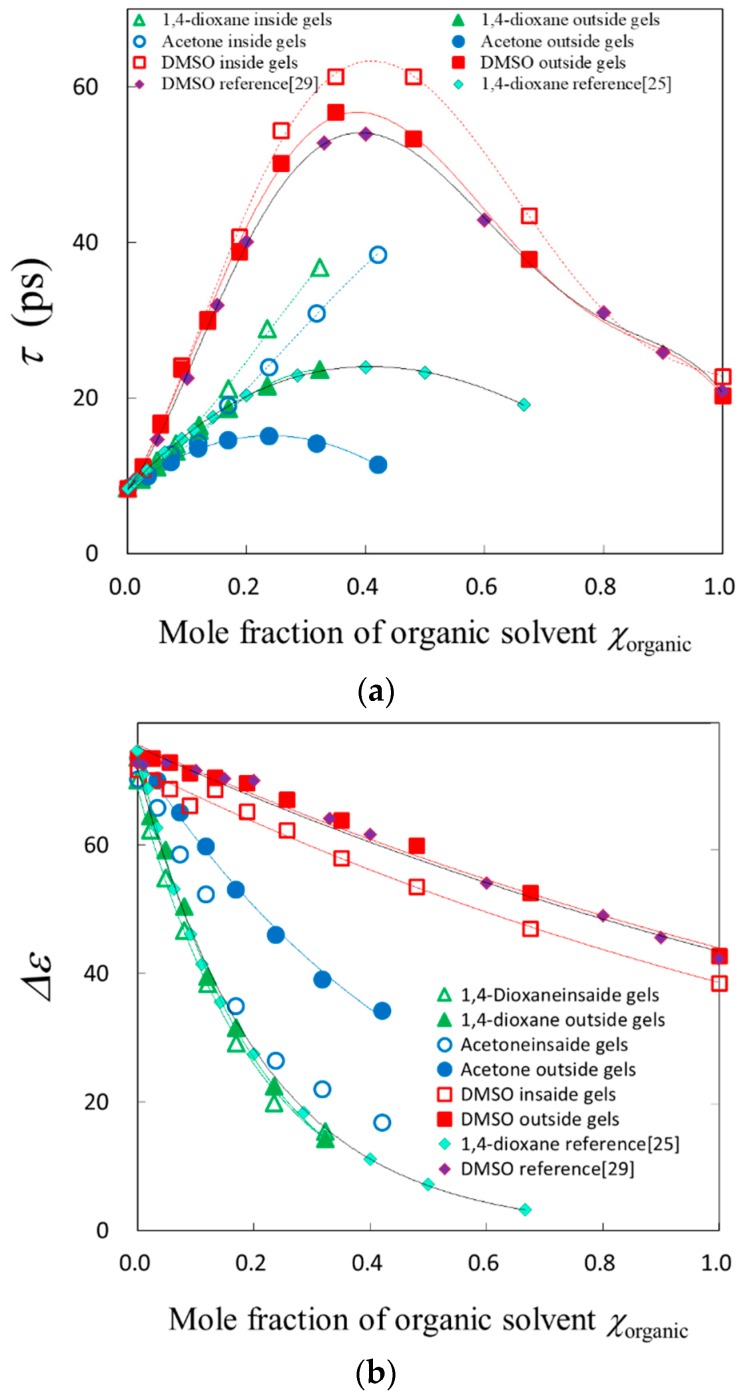
Composition dependences of the relaxation parameters: (**a**) Relaxation time; (**b**) Relaxation strength.

Figure 6 shows composition dependences of the relaxation time and the relaxation strength. The larger relaxation time corresponds to the large frequency shift to the lower frequency side. The relaxation strength was smaller for the solvent inside gels because of the decrease in the density of solvent molecules.

**Figure 7:**
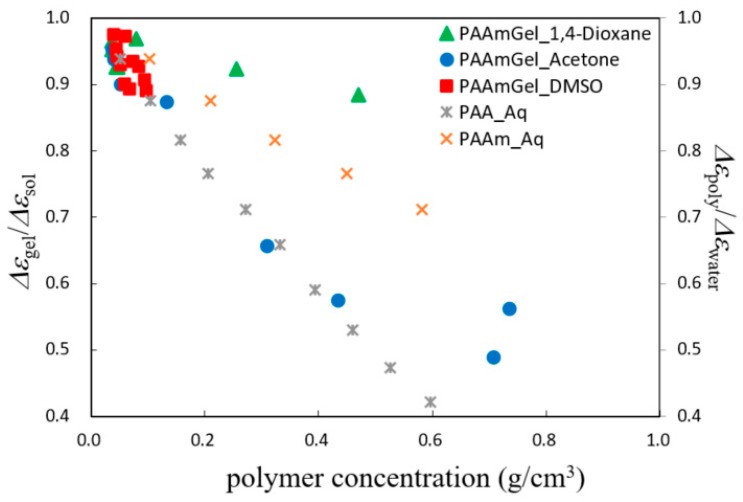
Polymer concentration dependence of the normalized relaxation strength for each solvent mixture.

Figure 7 shows polymer concentration dependence of the normalized relaxation strength for each solvent mixture. The normalized relaxation strength for aqueous solutions of PAAm and poly (acryl acid) (PAA) were also shown for comparison. DMSO aqueous solutions kept larger values, since the gel did not shrink enough even at low water content. Results for acetone- and 1,4-dioxane-water mixtures show similar dependency of decreasing swelling ratio, but the values of normalized relaxation strength for 1,4-dioxane–water mixtures were larger than those for acetone–water solutions, since the 1,4-dioxane is a non-polar organic solvent. Furthermore, the polymer concentration dependence of the normalized relaxation strengths for acetone aqueous solutions showed similar values to those shown for water in PAA aqueous solution and the same kind of behavior as those for PAA and PAAm aqueous solutions in the polymer concentration region less than 0.6, but the dependency exhibited a different manner in the region above 0.6. This result suggests the existence of another phenomenon in collapsed gels.

**Figure 8:**
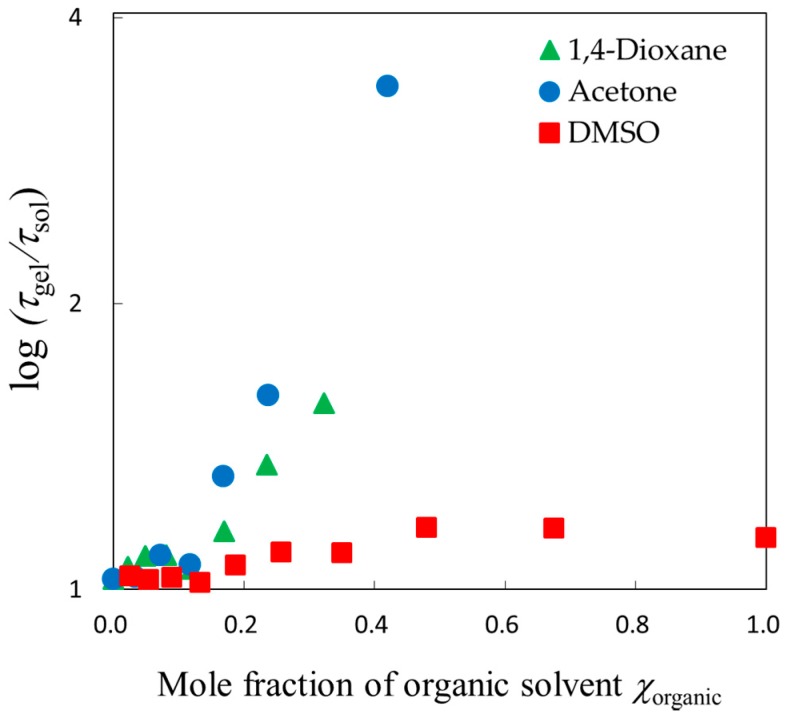
Composition dependences of the relaxation time; log plot of the normalized relaxation time against the mole fraction of organic solvent of the aqueous solutions.

The relaxation time for solvent mixtures in gels normalized by those outside gels are plotted against the composition of each solvent mixture in Figure 8. Characteristic behaviors were shown for the normalized relaxation time in the mole fraction region of 0.1 < *χ*_organic_ < 0.2, in which the swelling ratio abruptly changed. Aqueous molecular liquids apparently show a discontinuous behavior around a characteristic mole fraction of water, 83%, though Buchner et al. reported that there exists no corresponding characteristic liquid structure [31]. In the mole fraction region of 0.2 < *χ*_organic_ < 0.5, the normalized relaxation time for each solvent mixture seems to increase simply with shrinking gel. The normalized relaxation parameters reflect the swelling ratio well. 

Composition dependence of the normalized relaxation time is shown in Figure 8, the normalized relaxation time reflects much more restrictions from the polymer network through the swelling ratio. Considering that the chain dynamics are slower than those of solvent molecules in the present study, it is reasonable that slow dynamics of solvent molecules are determined by chain dynamics of low mobility polymer networks more than solvent dynamics with high mobility.

**Figure 9:**
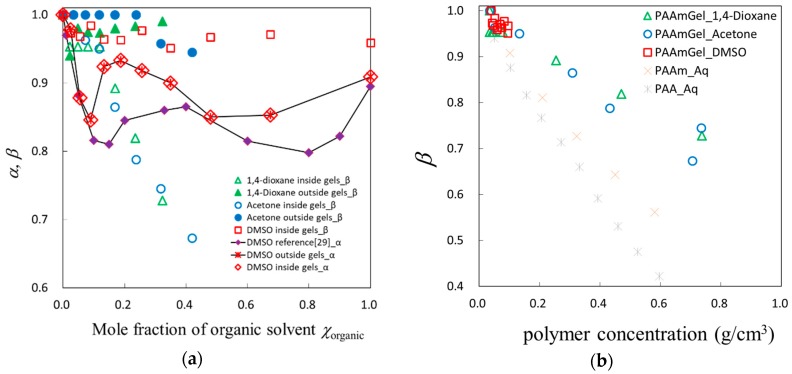
Behaviors of the relaxation time distribution parameter: (**a**) Composition dependence of the relaxation time distribution parameters, *α* and *β*, for solvent mixtures inside or outside the gels; (**b**) Polymer concentration dependence of the relaxation time distribution parameter, *β*, for solvent mixtures inside.

The relaxation time for the dipole relaxation process is a characteristic time of molecular dynamics of the rotational diffusion, but it is just a measure of the average value. Then, the distribution of the relaxation time is necessary for more exact analysis and detailed discussion. Figure 9a shows composition dependence of the relaxation time distribution parameters, *α* and *β*, defined by Equation (1), and the *β* values are plotted against the polymer concentration in Figure 9b. The parameters, *α* and *β*, are related to asymmetric and symmetric broadening of the relaxation curve [31]. The physical meanings of the parameters, *α* and *β*, are molecular interactions similar to the chain connectivity of polymers and fractal fluctuations of density, respectively [32]. Then, parameters *α* and *β* can be respectively treated even in the case of relaxation processes described by the Havriliak–Negami Equation. Figure 9b shows similar broadening for three organic solvent mixtures with increasing restriction of shrinking gels in the concentration region between 0.1 and 0.6, except for the characteristic behaviors shown at concentration below 0.1. In the high concentration region above 0.6, no clear concentration dependence is shown. This result suggests a possible explanation in which the composition of the solvent mixtures inside the gel is different from that outside the gel. The organic solvent concentration in the gel is considered to be lower than that expected at the same composition of solvent mixtures existing outside gel, since PAAm chains are hydrophilic.

**Figure 10:**
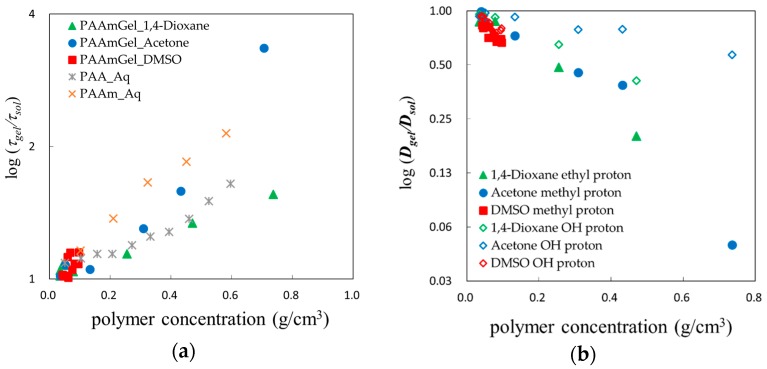
Polymer concentration dependences of the relaxation time and diffusion coefficient for solvent molecules restricted in polymer chain networks: (**a**) Logarithm of the normalized relaxation time; (**b**) Logarithm of the normalized diffusion coefficient.

Logarithm of the normalized relaxation time, *τ_gel_*/*τ_sol_*, and the normalized diffusion coefficient, *D_gel_*/*D_sol_*, are plotted against polymer concentration in Figure 10a,b. Polymer concentration dependence of the relaxation time and the diffusion coefficient show reasonable tendencies, respectively, reflecting restrictions from shrinking polymer networks except for the low polymer concentration region less than 0.1 g/cm^3^, in which characteristic behaviors are shown.

### 2.4. Scaling Concepts

The interactions among mixed solvents and polymer chains in gels clearly showed characteristic features of those dynamic properties. Though the dynamic properties of solvent mixtures in gels were normalized by those in bulk solvent mixtures, each system still shows characteristic behavior. Scaling concepts were used to examine the universal property of the restrictions.

Solvent dynamics restricted by shrinking gels have been analyzed by scaling law, especially for translational diffusion constants obtained from PFG-NMR measurements for probe or solvent molecules [9,10,33]. To investigate the molecular dynamics of solvent restricted in polymer networks, the scaling variable expressed by a ratio of sizes of the solvent molecule and the mesh size of the polymer networks can suggest a physical picture of the molecular mechanism, as

*x* = *R*/*ξ* (2)

where the scaling variable, *x*, is expressed as a ratio of sizes of the solvent molecule, *R*, and the mesh size, *ξ*, of the polymer chain networks. Following power law relationship, *R* and *ξ* are rewritten by the molecular weight of solvent molecules, *M*, and polymer concentration, *C_p_* [34] as

(3) R∝M13, ξ∝Cp−34

Using Equation (3) and suitable exponents, the normalized relaxation time, *τ_gel_*_/_*τ_sol_*, and the diffusion coefficient, *D_gel_*/*D_sol_*, were finally expressed by

(4) τgelτsol∝f(x3)=f{(Rξ)3}=exp{(M13Cp34)3}

and

(5) DgelDsol∝f(x−1)=f{(Rξ)−1}=exp{(M13Cp34)−1}

Here, the scaling exponents were three and unity, respectively, for Equations (4) and (5). The *M* values used for the scaling analysis with Equation (5) were determined as those for the probe of solvent molecules. On the other hand, the *M* values used for the scaling analysis with Equation (4) were determined as averages following the composition of water and organic solvent molecules, since only a single cooperative relaxation process is observed for the solvent mixtures. Other values of scaling exponent could not represent straight lines. These analyses of the scaling law suggest that the scaling variable *x* (=*M*^1/3^*C_p_*^3/4^) is available for the normalized diffusion coefficient *D_gel_*/*D_sol_* obtained by the PFG-NMR method and *x*^3^ (=*MC_p_*^9/4^) for the normalized relaxation time, *τ_gel_*_/_*τ_sol_* obtained by the TDR method.

**Figure 11:**
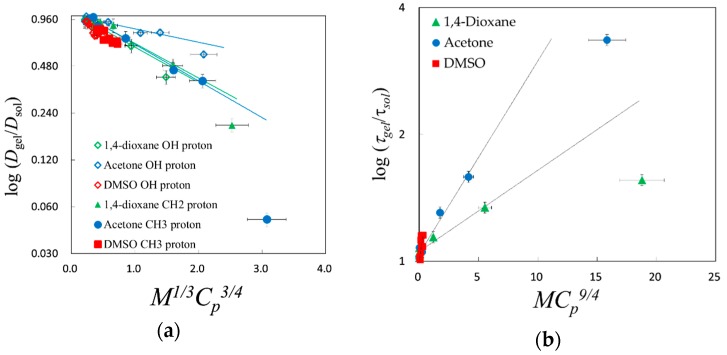
This is a figure, Schemes follow the same formatting. If there are multiple panels, they should be listed as: (**a**) Description of what is contained in the first panel; (**b**) Description of what is contained in the second panel. Figures should be placed in the main text near to the first time they are cited. A caption on a single line should be centered.

Figure 11a,b finally shows linear relationships if the plot for the most collapsed gel for the acetone aqueous solution is neglected. Error bars shown in Figure 11 express the accuracy reflecting volume, NMR, and dielectric measurements. These obvious differences for the plot for the most collapsed gel for acetone aqueous solution were also shown in Figure 7 and Figure 9b. Considering the effect of the existence of a small amount of water molecules remaining in the most collapsed gel with the hydrophilic properties of PAAm, the plot shifts to the larger value of *D_gel_*/*D_sol_* and the smaller value of *τ_gel_*_/_*τ_sol_*, respectively, in Figure 11a,b. These compensations tend to return those plots to straight lines.

The result of different scaling exponents obtained for Equations (4) and (5) is supposed to reflect the difference in the physical meanings of the relaxation time and the diffusion coefficient. Both physical properties related to diffusive dynamics of molecules in the medium are expressed with friction described by the Stokes law, as

(6) $$D=\frac{\mathrm{kT}}{6\pi \eta r}$$

and

(7) $$\tau_{rot}=\frac{V\eta }{\mathrm{kT}}$$

here k is the Boltzmann constant, T is the absolute temperature, *r* and *V* are the radius and the volume of molecules, respectively. Equation (7) implies that the relaxation time is determined by the intermolecular interactions expressed by the ratio of volumes for the solvent molecule and the mesh of polymer network. Therefore, this expression for the relaxation time seems to be reasonable, since typical expression of dynamic behaviors are often treated with the free volume theory.

### 2.5. Fractal Analysis with τ–β Diagram

Recently, we have examined fractal analysis for evaluation of the water structure [35,36,37]. Ryabov et al. expressed the relationship between the Cole–Cole relaxation time distribution parameter and the relaxation time [38,39] as

(8) $$\beta =\frac{d_{G}}{2}\frac{\ln\left(\tau \omega_{s}\right)}{\ln\left(\tau /\tau_{0}\right)}$$

where *τ*_0_ is the cutoff time of the scaling in time domain, *d_G_* is the fractal dimension of the point set where relaxing units are interacting with the statistical reservoir,

(9) $$\omega_{s}=2d_{E}G^{2/d_{G}}D_{s}/R_{0}{}^{2}$$

is the characteristic frequency of the self-diffusion process where *d_E_* is the Euclidean dimension, *D_s_* is the self-diffusion coefficient, *R*_0_ is the cutoff size of the scaling in the space, and *G* is a geometrical coefficient approximately equal to unity. This analysis requires a fractal dimension to combine both the average value of characteristic time of dynamics (the relaxation time) and the fluctuation (Cole–Cole relaxation time distribution parameter), and it is not necessary to know any exact values of the water content. Finally, we know how water molecules aggregate and disperse in materials from the fractal analysis.

**Figure 12:**
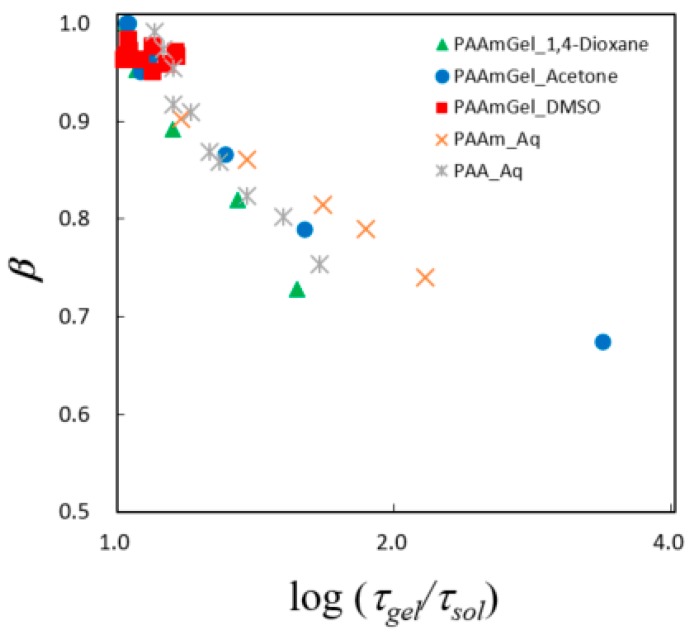
Plot of the logarithm of the relaxation time distribution parameter of Cole–Cole function against the normalized relaxation time, *τ_g__el_*/*τ_sol_*.

Equation (8) was examined for the GHz frequency process observed in the present work. Generally, the fractal analysis requires plotting of the Cole–Cole relaxation time distribution parameter against the logarithm of the relaxation time, and obtained hyperbolic curve is analyzed. In the present work, the normalized relaxation time, *τ_gel_*/*τ_sol_*, was used for the analysis, since slow dynamics due to restriction from shrinkage of the polymer network were treated. Figure 12 shows the *τ–β* diagram for the present PAAm gels with organic solvent aqueous solutions. The plots obtained for the solvent molecules restricted in polymer chains show hyperbolic curves for aqueous solutions. Usually, plots for gels are located in the region lower than those for solutions in the Figure, and the water structures in gels are more heterogeneous than those for solutions. In the present work, however, the curves obtained for organic solvent aqueous solutions restricted in PAAm gels take similar shape and occur in a slightly lower region compared to curves for aqueous solutions of PAAm and PAA. The plot for the collapsed gel occurs in the upper right region because of the under-estimation of *τ_sol_* for the remaining water molecules. This tendency means that the mesh size of the polymer network cannot be more homogeneous than the polymer chains in the solutions, and this result follows the characteristic behaviors of gel, solution, and dispersion systems which we have obtained in recent works [35,37,40].

The fractal analysis performed for the GHz frequency process with 10 ps time scale obtained from TDR measurements cannot be treated in the same manner as NMR measurements with 1 ms time scale. The dynamic properties of cooperative interactions of hydrogen bonding networks treated by TDR measurements were not reflected in larger scale observation of diffusion coefficients caused by the averaging effect [37]. The fractal analysis used in dielectric study is remarkably useful for evaluation of water structures, especially for investigating how water molecules are coagulated and dispersed.

## 3. Conclusions

Restrictions of solvent molecule dynamics in PAAm gels were analyzed by TDR and PFG-NMR measurements for mixed solvents of acetone–, 1,4-dioxane–, and DMSO–water. The restrictions could be expressed by the scaling law with a scaling variable of the ratio between the size of solvent molecules and the mesh size of the PAAm network. Suitable exponents were determined as unity and three for the diffusion coefficient and the relaxation time, respectively. The fractal analysis suggests that the water structure of polymer networks cannot be more homogeneous than those in polymer solutions.

## 4. Materials and Methods

### 4.1. Materials

The preparation of the poly (acrylamide) (PAAm) gel was following Tanaka et al. [1]. Acrylamide (5 g), *N*,*N*’-methylene-bis-acrylamide (0.133 g), ammonium persulfate (40 mg), and *N*,*N*, *N*,*N*-tetra-methylethylene-diamin (TEMED) (240 μL) were dissolved in distilled and deionized water (milli-Q system: Merck Millipore Japan Co., Ltd., Tokyo, Japan) to a final volume of 100 mL. The solution was poured into glass tubes with a diameter of 7 mm. It appears to take approximately 2 h for gelation at room temperature. The gel was cut in the 10 mm column and immersed in deionized water for 3 days to wash away residual acrylamide, bis-acrylamide, ammonium persulfate, and TEMED. The volume of the gels decreased with increasing the organic solvent composition and volume phase transitions were observed. The aqueous solution of monomer, initiator, and reaction accelerator was prepared and was put into a glass tube (length: 50 mm, diameter: 8 mm) and was kept for 2 h. The gel was cut in the 10 mm column and immersed in pure water for 2 days to wash away residual acrylamide, bis-acrylamide, and other impurities. The gels were immersed in the solvent of various compositions. 

Acetone, 1,4-dioxane, and dimethyl sulfoxide (DMSO) were used as organic solvent to decrease the volume of the gels. The gels were placed in acetone–water and 1,4-dioxane–water mixtures with those concentrations from 0 to 70 wt % at 10 wt % intervals. In addition, DMSO–water mixtures with those concentrations from 0 to 100 wt % at same intervals.

In NMR measurements, the samples were cut out by cover glass and put into the aspirator tube with outer diameter: 2.0 mm and the inner diameter: 1.4 mm. In the case of acetone and 1,4-dioxane aqueous solutions, the size of samples obtained for more than 70 wt % organic solvent were too small to make measurements.

### 4.2. Experimental Methods

#### 4.2.1. Volume, Density, and Viscosity

The volume of the gel was calculated from diameter and length determined by caliper. The diameter and length were averages of 5 times measurements. Density measurements for 10 wt % organic solvent–water mixtures were performed at 25 °C by density meter DMA48 (Anton Paar, Tokyo, Japan).

#### 4.2.2. NMR Measurements

**Figure 13:**
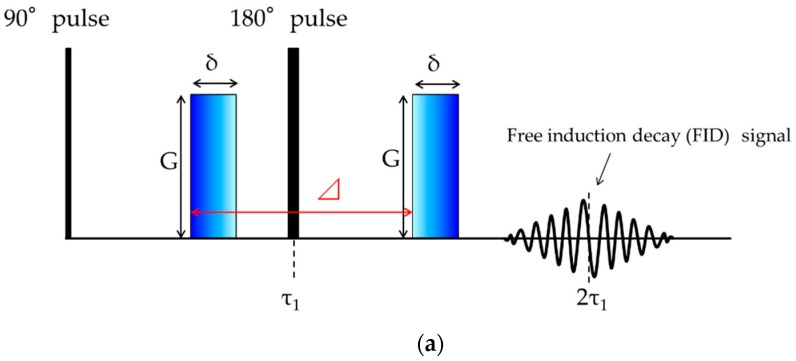

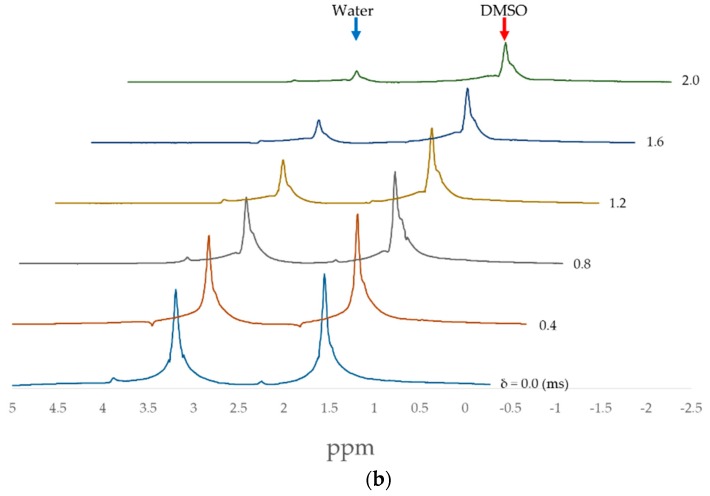
(**a**) The pulse sequence for the spin-echo method for measurement of diffusion coefficient by pulsed field gradient. Block pulse written by a diagonal gradation is a magnetic gradient; (**b**) The spin echo signal attenuation of 1 H NMR spectra for 60 wt % DMSO aqueous solutions inside gels by varying field gradient pulse duration, *δ*.

Nuclear magnetic resonance (NMR) was used to determine the diffusion coefficient. The experiments were performed on a nuclear magnetic resonance spectrometer (EX-90, JEOL, Tokyo, Japan), which was equipped with a pulsed field gradient spin echo (PFG-SE). Figure 13a shows the pulse sequence used for PFG-SE NMR measurements. The principles of the PFG-SE technique have already been reported in detail [32]. For the calibration of the gradient magnetic field strength, the diffusion coefficient of Reference [41] was used. The temperature was controlled to 25.0 ± 0.2 °C. The diffusion coefficients of the probe molecules are determined from the intensity of spin echo signal [7]. The intensity of the spin echo signal, *A*, in the presence of the field gradient pulses is expressed as follows:

(10) $$A=A_{0}exp\left[-\gamma^{2}\delta^{2}G^{2}\left(\Delta -\frac{\delta }{3}\right)D\right]$$

here, *A*_0_ is the echo amplitude in absence of the field gradient pulses, *γ* is the magnetogyric ratio of the observed nucleus, *δ* is the duration of the field gradient pulse, 0.1~2.0 ms. *G* is the intensity of pulse field gradient, 87 gauss/cm. Δ is the time interval, 20 ms, between the leading edges of the field gradient pulses, and *D* is the diffusion coefficient of the probe molecule, respectively.

Figure 13b shows an example of spin echo signal attenuation of two peaks, respectively, for water and DMSO protons obtained at every 0.4 ms of *δ* between 0 and 2.0 ms. Each *D* value was obtained from fitting procedures of the attenuation of normalized amplitude obtained at every 0.1 ms of *δ* between 0.3–1.3 ms with Equation (10).

#### 4.2.3. Dielectric Measurements

TDR measurements were performed by digitizing oscilloscope (HP54120B, Agilent Technology, Tokyo, Japan) and Four Channel Test Set (HP54124A, Agilent Technology) with a homemade open-end coaxial electrode with an outer diameter of 2.2 mm. Contact of the open-end of the electrodes to the surface of the gels offers a fringing field penetrating inside the gel and practical dielectric measurements. Dielectric measurements of the solution were performed for solvent outside the gel. Applied voltage was 200 mV and time ranges used were 50, 100, 200, 500, and 1000 ps/div. Temperature was controlled by a homemade temperature jacket at 25.0 ± 0.5 °C.
